# MdHIR proteins repress anthocyanin accumulation by interacting with the MdJAZ2 protein to inhibit its degradation in apples

**DOI:** 10.1038/srep44484

**Published:** 2017-03-20

**Authors:** Ke-Qin Chen, Xian-Yan Zhao, Xiu-Hong An, Yi Tian, Dan-Dan Liu, Chun-Xiang You, Yu-Jin Hao

**Affiliations:** 1 National Key Laboratory of Crop Biology, College of Horticulture Science and Engineering, Shandong Agricultural University, Tai-An, Shandong 271018, China; 2 State Key Laboratory of Crop Stress Biology for Arid Areas, College of Horticulture, Northwest A&F University, Yangling, Shaanxi 712100, China

## Abstract

In higher plants, jasmonate ZIM-domain (JAZ) proteins negatively regulate the biosynthesis of anthocyanins by interacting with *bHLH* transcription factors. However, it is largely unknown if and how other regulators are involved in this process. In this study, the apple MdJAZ2 protein was characterized in regards to its function in the negative regulation of anthocyanin accumulation and peel coloration. MdJAZ2 was used as a bait to screen a cDNA library using the yeast two-hybrid method. The hypersensitive induced reaction (HIR) proteins, MdHIR2 and MdHIR4, were obtained from this yeast two-hybrid. The ZIM domain of MdJAZ2 and the PHB domain of the MdHIR proteins are necessary for their interactions. The interactions were further verified using an *in vitro* pull-down assay. Subsequently, immunoblotting assays demonstrated that MdHIR4 enhanced the stability of the MdJAZ2-GUS protein. Finally, a viral vector-based transformation method showed that MdHIR4 inhibited anthocyanin accumulation and fruit coloration in apple by modulating the expression of genes associated with anthocyanin biosynthesis.

Fruit color is often a main indicator used to evaluate the fruit’s economic value. In many fruits, such as apple, grapevine and peach, anthocyanins play a crucial role in the coloration of the peel and the flesh. Anthocyanins are derivatives of glucosides. They belong to the flavonoid compound family and ubiquitously exist in the vacuole of cells in the flower, fruit, root, stem, and leaf. There are generally six anthocyanin pigments, cyanidin, delphinidin, pelargonidin, peonidin, petunidin and malvidin, found in fruits. Cyanidin is the most common pigment and is produced in over 82% of researched fruits and berries^1^. Delphinidin and its methylated derivatives, such as petunidins and malvidins, appear as dark bluish and purple colors, but cyanidins and pelargonidins are the main pigments in bright red-colored fruits^1^. There are other factors, such as co-pigmentation and pH, which affect the perceived hue of a tissue^2^.

In higher plants, including apple and other fruit trees, the main biosynthesis pathway of anthocyanins is the flavonoid pathway^3^. The synthetic enzymes and their encoding genes, also called structural genes, have already been extensively identified and investigated^4^. As is well known, the expression of the structural genes are regulated by various transcription factors (TFs) such as HY5 and MYB-bHLH-WD40 (MBW)^5^,^6^,^7^.

MYB TFs are the most intensively studied components of the MBW complex associated with anthocyanin biosynthesis in fruit trees^8^,^9^,^10^. In apple, *MdMYB10, MdMYB1, MdMYB9, MdMYB11, MdMYB110a, MdMYB3* and *MdMYBA* activate the expression of the anthocyanin structural genes, while *MdMYB15, MdMYB16, MdMYB17, MdMYB27, MdMYB28, MdMYB49, MdMYB50* and *MdMYB111* repress them^10^,^11^,^12^,^13^,^14^,^15^. Furthermore, *MdMYB1* directly regulates the expression of the *MdDFR* and *MdUFGT* genes^16^. In addition to the MYB TFs, another type of MBW TFs, known as bHLH or MYC, are involved in the regulation of the anthocyanin structural genes. *MdbHLH3* and *MdbHLH33* not only directly regulate the expression of the structural genes but also bind to the promoters of MYB genes, such as *MdMYB1, MdMYB9, MdMYB10, MdMYB11*, and modulate their transcription^11^,^12^,^15^. A WD repeat protein, MdTTG1, which is homologous to *Arabidopsis* TRANSPARENT TESTA GLABRA1, is also involved in the regulation of anthocyanin accumulation in apple^17^,^18^.

Various environmental factors, such as light and temperature, influence the biosynthesis of anthocyanins^19^. The MBW complex also works as regulatory machinery for anthocyanin accumulation in response to various developmental and environmental cues. In apple, MdMYB1 and MdbHLH3, MBW components, are regulated at transcriptional and posttranslational levels to modulate anthocyanin accumulation in response to light and temperature^12^,^20^. In addition, plant hormones such as auxin, ethylene, gibberellin, jasmonic acid (JA), cytokinins and abscisic acid modulate anthocyanin synthesis^21^,^22^,^23^. Jasmonate ZIM-domain (JAZ) proteins are the key regulators in the JA signaling pathway^24^. In the classic *Arabidopsis* model of the JA signaling pathway, when the JA level is relatively low JAZ proteins accumulate and interact with the bHLH TF *MYC2*, consequently attenuating the expression of the downstream JA-responsive genes^25^. When the JA level is relatively higher, however, the receptor SCF-COI1 complex binds with the JAZ protein to form the new SCF-COI1-JAZ complex^26^. The JAZ protein is then ubiquitinated by E3 ubiquitin ligase and degraded by 26S proteasome^27^. Finally, JA responsive genes are transcribed^28^,^29^.

In plants, JAs are crucial hormones involved in plant development and growth, secondary metabolism and defense against biotic and abiotic stresses^30^. The JAZ proteins play a principal role in various functions of JA. For example, they interact with the R2R3-MYB TFs, such as *MYB21* and *MYB24*, to affect JA-regulated stamen development in *Arabidopsis*^31^. They also interact with the bHLH TFs, *MYC2, MYC3* and *MYC4*, to repress JA-induced defense against bacterial pathogens and insect herbivory^32^.

In *Arabidopsis*, the JAs also induce anthocyanin biosynthesis by up-regulating TF genes, such as *PAP1, PAP2* and *GL3*, which modulate the expression of the anthocyanin biosynthetic genes *DFR, LDOX* and *UF3GT*^33^. Furthermore, the JAZ proteins interact with the bHLHs, such as GL3, EGL3, and TT8, and with the MYB TFs, such as MYB75 and GL1, which regulate the expression of the MBW complexes associated with anthocyanin biosynthesis, attenuating their transcriptional function and, thereby, inhibiting anthocyanin biosynthesis^34^. In apple, MdbHLH3 promotes anthocyanin biosynthesis by directly upregulating the expression of not only structural genes, such as *MdDFR* and *MdUFGT* but also regulatory MYB genes, including *MdMYB1, MdMYB9* and *MdMYB11*. Additionally, MdJAZ2 interacts with MdbHLH3 and inhibits its transcriptional function, thereby, working as a negative regulator for anthocyanin biosynthesis^12^,^15^.

In *Arabidopsis*, a few of proteins are characterized by their interaction with the JAZ proteins, which influences their functions. DELLA proteins compete with MYC2 for binding to the JAZs to promote the expression of downstream genes^35^. JAZ proteins directly interact with EIN3/EIL1 and recruit HDA6 to suppress the transcriptional activity of EIN3/EIL1 to regulate plant development and defense responses^36^. In this study, a yeast two hybridization approach was conducted using MdJAZ2 as bait to screen through an apple fruit peel cDNA library. Hypersensitive induced reaction (HIR) protein, MdHIR2 and MdHIR4, were found to interact with MdJAZ2. The interaction of the MdHIRs with the MdJAZ proteins and their function in the regulation of anthocyanin biosynthesis was verified and characterized. Our findings provide evidence that the MdHIRs acts upstream to the MdJAZ2-MdbHLH3 pathway to negatively regulate anthocyanin accumulation in apple.

## Results

### MdJAZ2 inhibits anthocyanin accumulation in apple peel

**Figure 1:**
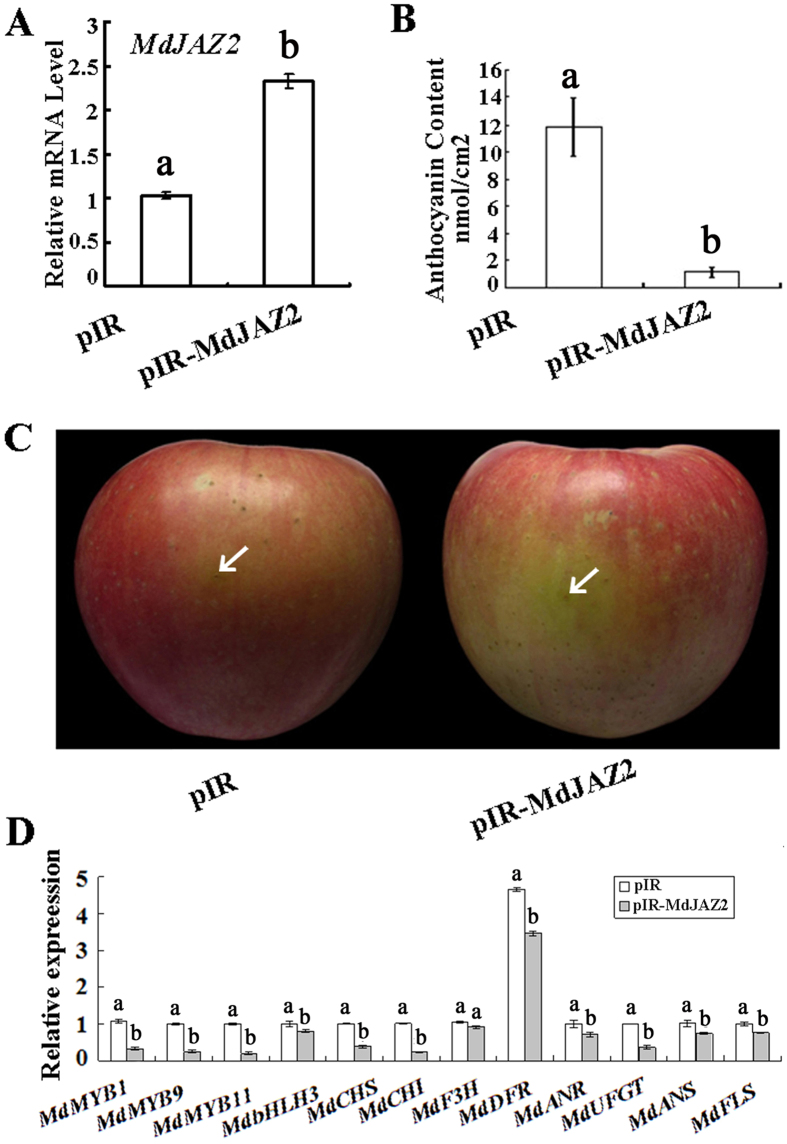
MdJAZ2 inhibits anthocyanin accumulation in the peel of the apple fruit. (**A**) Quantitative real-time PCR analysis of the expression of *MdJAZ2* in the fruit peel surrounding the injection sites. (**B**) The anthocyanin content of the fruit peel around the injection sites. Anthocyanins were extracted from 8 pieces fruit skin, 1 cm^2^ in size for each from one individual fruit. (**C**) The phenotype of the peel surrounding the injection sites. The apple fruit were injected with a viral based overexpression vector, pIR-*MdJAZ2*, while the empty vector, pIR, was used as the control. The injected apples were kept in an illumination incubator under white light at 17 °C for 4 days. (**D**) Quantitative real-time PCR analysis of the regulatory and structural genes associated with anthocyanin biosynthesis in the fruit peel around the injection sites. The data are shown as the mean ± SE, which were calculated based on 3 replicates. Mean differences in the bars are significant at *P*_*0.05*_ level with different letters, not significant at *P*_*0.05*_ level with the same letters. Repeat in following figure.

To examine if MdJAZ2 influences anthocyanin accumulation and peel coloration in apple fruit, a viral vector-based transient transformation method was conducted to enhance the expression level of *MdJAZ2* in the apple peel^20^. The viral overexpression vector pIR*-MdJAZ2,* plus a helper plasmid, IL-60-BS, were injected into the fruit peel of cultivar ‘Red delicious,’ while the empty vector pIR, plus IL-60-BS, served as the control. The expression analysis demonstrated that pIR-MdJAZ2 injection noticeably enhanced the expression of *MdJAZ2* gene in apple fruit peel than the pIR control (Fig. 1A). Subsequently, anthocyanin content was measured in the fruit peel around the injection sites. Anthocyanin content in the apple peel injected with pIR*-MdJAZ2* was much lower than in the apple peel injected with the empty control (Fig. 1B). As a result, the injection of pIR*-MdJAZ2* resulted in a loss of red colouration in the apple skin, compared with the empty control (Fig. 1C).

We previously reported that MdJAZ2 interacts with MdbHLH3 and inhibits the expression of *MdMYB1, MdMYB9, MdMYB11* and their downstream anthocyanin structural target genes^15^. Therefore, the transcript levels of these genes were examined with real-time quantitative RT-PCR in the apple peel around the injection sites. The results indicated that *MdJAZ2* transient overexpression slightly influenced the expression of *MdbHLH3*, but significantly repressed the transcript levels of *MdMYB1, MdMYB9* and *MdMYB11* (Fig. 1D). Consequently, the reduced expression of these genes repressed the expression levels of the anthocyanin structural genes to different degrees. Affected genes included *MdCHS, MdCHI, MdANR, MdDFR, MdUFGT, MdF3H, MdANS* and *MdFLS* (Fig. 1D).

Therefore, MdJAZ2 functions as a negative regulator of anthocyanin accumulation and fruit coloration by repressing the expression of the anthocyanin regulatory and structural genes in apple.

### Yeast two-hybrid (Y2H) screening of an apple peel cDNA library reveals a putative MdJAZ2-interacting protein, MdHIR2

**Figure 2:**
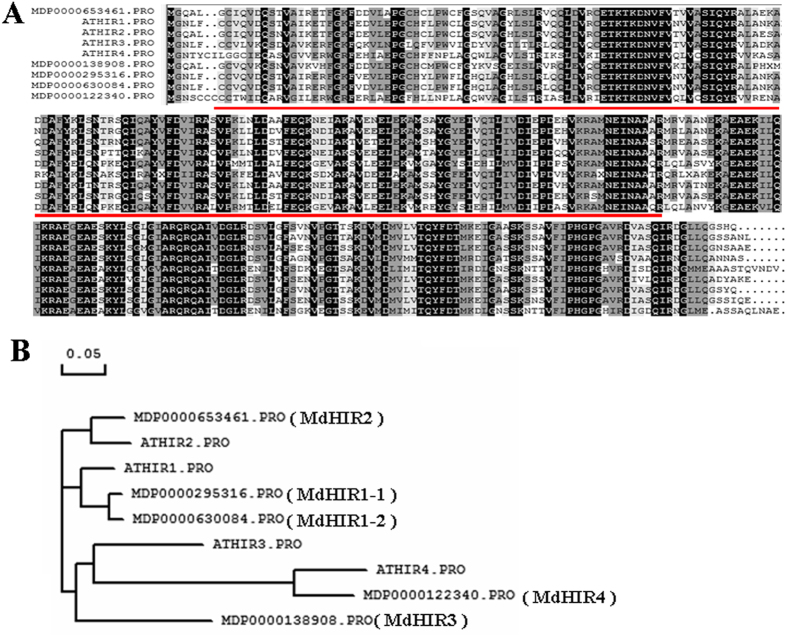
Sequence analysis of the HIR proteins in apple and *Arabidopsis*. (**A**) Comparison of the predicted amino acid sequences of the HIR proteins in apple and *Arabidopsis*. The amino acid consensus sequences are highlighted in black. Md, *Malus domestica*; At, *Arabidopsis thaliana* (AtHIR1, AT5G62740; AtHIR2, AT1G69840; AtHIR3, AT3G01290; AtHIR4, AT5G51570). The HIR proteins contained highly conserved PHB (prohibitin homologues) domains, which are indicated as a red line. (**B**) A phylogenetic tree of the HIR proteins in *Malus domestica* and *Arabidopsis*.

Y2H screening was conducted to screen MdJAZ2-interacting proteins. As a result, from a library with a primary library titer of 8.16 × 10^6^ cfu/mL of which 95% of the clones had inserts, five positive colonies were obtained. Among the positive colonies a target cDNA was found that corresponds to the gene *MDP0000653461*.This gene encodes a putative protein that is similar to the hypersensitive induced reaction (HIR) proteins in *Arabidopsis*. There are a total of four HIR genes in *Arabidopsis, AtHIR1, AtHIR2, AtHIR3* and *AtHIR4*. In addition to *MDP0000653461*, four more *MdHIR* genes were found in the apple genome, *MDP0000295316, MDP0000630084, MDP0000138908* and *MDP0000122340*. Reverse Transcription PCR (RT-PCR) and sequence analysis demonstrated that all five apple *MdHIR* genes transcribed cDNAs that encode putative MdHIR proteins (Fig. S1). The predicted MdHIR proteins were used for sequence alignment analysis with AtHIRs. The sequence alignment analysis showed that the five predicted MdHIR proteins are highly similar in amino acid sequence to the AtHIRs (Fig. 2A).

In addition, protein domain searches were performed using the programs PFAM and SMART. These searches found that the five predicted MdHIR proteins contain an SPFH/Band7/PHB domain, which is highly conserved in the four AtHIRs (Fig. 2A). Subsequently, a phylogenetic tree was constructed using the four AtHIRs and the five MdHIRs. Based on the phylogenetic tree, the five MdHIRs were named MdHIR1–1 (MDP0000295316), MdHIR1-2 (MDP0000630084), MdHIR2 (MDP0000653461), MdHIR3 (MDP0000138908) and MdHIR4 (MDP0000122340) (Fig. 2B).

### MdHIR2 and MdHIR4 interact with MdJAZ proteins

**Figure 3:**
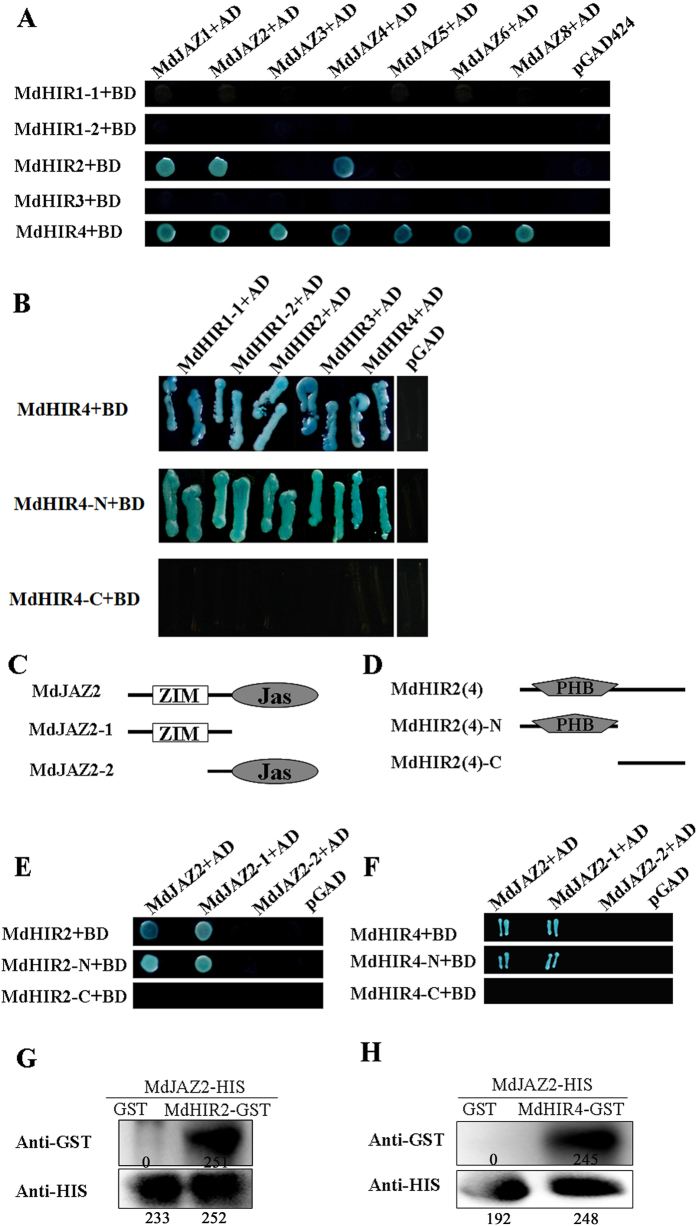
Interactions between the JAZs and the HIRs. **(A)** Yeast two-hybrid assays for the interactions between the MdJAZs and the MdHIRs. Seven apple MdJAZs were fused with the GAL4 activation domain (AD) in the pGAD424 vector, while 5 MdHIRs were fused with the GAL4 DNA binding domain (BD) in the pGBT9 vector. Interactions of the MdHIR proteins with the AD domain in the pGAD424 empty vector were used as the negative controls. Interactions are indicated by the blue color on SD/-Ade/-His/-Trp/-Leu/X-Gal medium. **(B)** Homo- and heterodimerization between the MdHIR4 protein, MdHIR4-N and the MdHIR1-1, MdHIR1-2, MdHIR2, MdHIR3 or MdHIR4 proteins. **(C)** Schematic diagram of the protein structure of MdJAZ2. The diagram shows the conserved ZIM and Jas domains. The domains found in MdJAZ2 were fused with the AD domain. **(D)** Schematic diagram of the protein structures for the MdHIR proteins. The diagram shows the conserved PHB domain. Different parts of MdHIR2 and MdHIR4 were fused with the BD domain. **(E,F)** Yeast two-hybrid assays of the interactions between the different domains of the MdHIR and MdJAZ2 proteins. **(G,H)** *In vitro* pull-down assays of the interaction of the MdJAZ2-His and MdHIR-GST proteins. Purified recombinant MdJAZ2-His protein was used to pull down MdHIR2-GST or MdHIR4-GST protein. The purified His tag was used to pull down the MdHIR-GST proteins as the negative control.

To verify the interaction between the MdHIRs and the MdJAZs, yeast two-hybrid assays were carried out. The full-length cDNA of each of the *MdHIR*s was inserted into the vector pGBT9 (BD*-MdHIRs*) as bait. The full-length CDSs of *MdJAZ1, MdJAZ2, MdJAZ3, MdJAZ4, MdJAZ5, MdJAZ6* and *MdJAZ8* were cloned with RT-PCR, and then inserted into the vector pGAD424 (AD*-MdJAZs*) as prey. Subsequently, each combination of the BD*-MdHIRs* and the AD*-MdJAZs* was co-expressed in yeast cells. The transformants were cultured on -Trp/-Leu/-His/-Ade screening medium and stained with X-α-gal. Yeast strains containing BD-*MdHIR2* plus AD-*MdJAZ1*, AD-*MdJAZ2* or AD-*MdJAZ4*, and BD-*MdHIR4* plus all seven of the AD-*MdJAZs* were positive for X-α-gal activity when grown on -Trp/-Leu/-His/-Ade screening medium. Yeast strains containing BD-*MdHIRs* or BD-*MdHIRs* plus the empty pGAD424 vector were negative for X-α-gal activity. Therefore, MdHIR2 interacted with MdJAZ1, MdJAZ2 and MdJAZ4, while MdHIR4 interacted with all seven of the MdJAZs tested (Fig. 3A). Interestingly, it was also found that MdHIR4 interacted with itself and four other MdHIR proteins (Fig. 3B).

To determine which region of the MdHIR proteins is necessary for interaction with the MdJAZ proteins, *MdHIR2* and *MdHIR4* were divided into the N-terminal PHB domain (*MdHIR2N, MdHIR4N*) and the C-terminus (*MdHIR2C, MdHIR4C*), while *MdJAZ2* was divided into the N-terminal ZIM domain (*MdJAZ2*ZIM) and the C-terminal Jas domain (*MdJAZ2*Jas). The corresponding cDNA fragments of *MdHIR2N* and *MdHIR2C* were inserted into pGBT9 (BD-*MdHIR2*N, BD-*MdHIR4*N and BD-*MdHIR2*C, BD-*MdHIR4*C) as baits, while *MdJAZ2*ZIM and *MdJAZ2*Jas were inserted into pGAD424 (AD-*MdJAZ2*ZIM and AD-*MdJAZ2*Jas) as preys. Yeast two-hybrid assays demonstrated that the N-terminal ZIM domain of the MdJAZ2 protein is necessary for its interaction with MdHIR2 and MdHIR4, while the N-terminal PHB domain of the MdHIR proteins is required for interaction with MdJAZ2 (Fig. 3C–F).

To further verify the interactions between MdHIR2, MdHIR4 and MdJAZ2 an *in vitro* pull-down assay was performed with MdHIR2-GST, MdHIR4-GST and MdJAZ2-HIS proteins that were expressed in and purified from the *Escherichia coli* strain BL21. The results showed that MdHIR2-GST and MdHIR4-GST, but not GST alone, interacted with the MdJAZ2-HIS protein (Fig. 3G,H).

Additionally, yeast two-hybrid and pull-down assays were also performed to examine the interactions between the four AtHIRs and the twelve AtJAZs in *Arabidopsis*. The yeast two-hybrid assays indicated that either AtHIR1 or AtHIR4 interacted with AtJAZ3, AtJAZ4 or AtJAZ9, while either AtHIR2 or AtHIR3 interacted with only AtJAZ3 (Fig. S2A). Subsequently, pull down assays confirmed the interaction between AtHIR1 and AtJAZ3 or AtJAZ9 (Fig. S2B,C).

### MdHIR4 enhances the stability of the MdJAZ2 protein in apple callus

**Figure 4:**
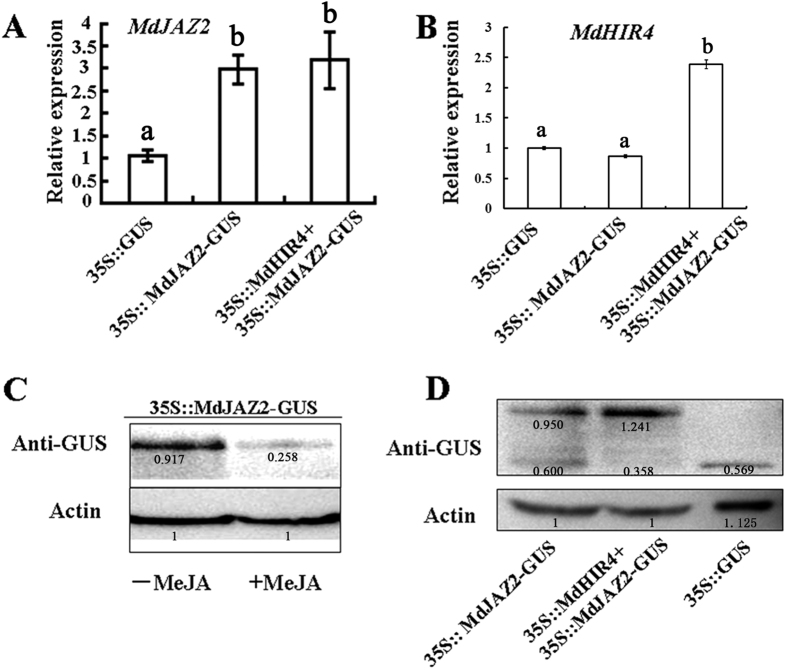
MdHIR4 enhances the stability of the MdJAZ2 protein in the ‘*Orin*’ callus. (**A,B)** Quantitative real-time PCR analysis of the relative expression of the *MdJAZ2* and *MdHIR4* genes in three transgenic apple calli, *35S::*GUS, 3*5S::MdJAZ2-GUS* and *35S::MdHIR4*+*35S::MdJAZ2-GUS*. **(C)** The immunoblotting assay using an anti-GUS antibody to determine the degradation of the MdJAZ2-GUS protein in the *35S::MdJAZ2-GUS* transgenic apple callus after being treated with or without 50 μM Me-JA. **(D)** The immunoblotting assay using an anti-GUS antibody to determine the abundance of the MdJAZ2-GUS and GUS proteins in the *35S::MdJAZ2-GUS, 35S::MdJAZ2-GUS*+*35S::MdHIR4* and *35S::GUS* transgenic ‘*Orin*’ calli.

To explore how MdHIR4 influences MdJAZ2, apple callus was transformed using an *Agrobacterium*-mediated method. Two constructs, pRI101-*35S::MdJAZ2*-*GUS* and pRI101-*35S::MdHIR4*, were made and used for transformation, while the empty vector, pRI101-*35S::GUS*, was used as a control. As a result, three types of transgenic apple calli, *35S::GUS, 35S::MdJAZ2-GUS* and *35S::MdJAZ2-GUS*+*35S::MdHIR4* were obtained. Quantitative real-time PCR was conducted to verify that the genes were expressed in the corresponding transgenic calli. The results showed that the *35S::MdJAZ2-GUS* transgenic callus generated more *MdJAZ2* transcripts, and *35S::MdJAZ2-GUS*+*35S::MdHIR4* transgenic callus exhibited higher levels of *MdJAZ2* and *MdHIR4* transcripts, than the *35S::GUS* control (Fig. 4A,B). In addition, the expression level of *MdJAZ2* was similar in *35S::MdJAZ2*-*GUS* and *35S::MdJAZ2-GUS*+*35S::MdHIR4* (Fig. 4A), and the expression level of MdHIR4 gene was similar in 35S::GUS and 35S::MdJAZ2 calli, indicating that *MdHIR4* and *MdJAZ2* did not influence each another at the transcriptional level (Fig. S3A).

Additionally, immunoblotting assays were conducted with an anti-GUS antibody to examine the abundance of MdJAZ2-GUS in the tested transgenic calli. The response of MdJAZ2-GUS to jasmonate signal was measured and indicated that MdJAZ2-GUS was degraded upon Me-JA treatment (Fig. 4C). The influence of MdHIR4 on the stability of MdJAZ2-GUS was examined. The results indicated that compared with the GUS protein in the *35S::GUS* control calli, both the *35S::MdJAZ2-GUS* and *35S::MdJAZ2-GUS*+*35S::MdHIR4* transgenic calli produced MdJAZ2-GUS, a fusion protein that has a higher molecular weight than GUS alone (Fig. 4D). In addition, there is a smear of proteins in the *35S::MdJAZ2-GUS* calli, which suggests a degradation of the larger molecular weight MdJAZ2-GUS fusion protein (Fig. 4D). However, there was almost no degradation product obviously found in the *35S::MdJAZ2-GUS*+*35S::MdHIR4* transgenic calli (Fig. 4C,D). This indicates that the *35S::MdJAZ2-GUS*+*35S::MdHIR4* transgenic calli accumulated more MdJAZ2-GUS protein than the *35S::JAZ2-GUS* transgenic calli, suggesting that MdHIR4 enhanced the stability of the MdJAZ2-GUS protein.

### MdHIR4 represses the accumulation of anthocyanins in the apple peel

**Figure 5:**
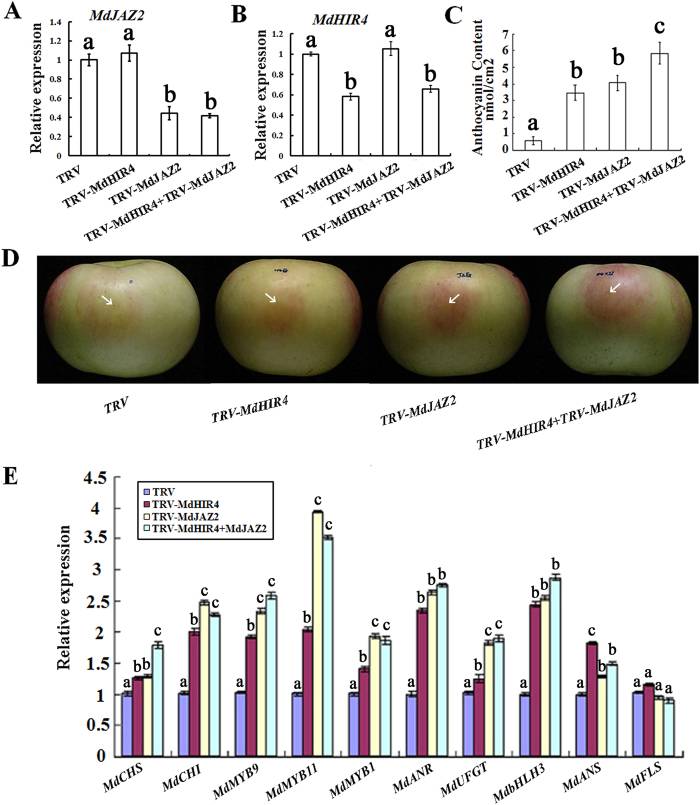
Suppression of the *MdHIR4* gene promotes anthocyanin accumulation in the peel of the apple fruit. **(A,B)** The expression levels of the *MdJAZ2* and *MdHIR4* genes in the fruit peel around the injection sites. **(C)** The anthocyanin content in the fruit peels around the injection sites. Anthocyanins were extracted from 8 pieces, 1 cm^2^ in size for each from one individual fruit. **(D)** Apple fruit coloration around the injection sites. Antisense *MdHIR4* and *MdJAZ2* cDNAs were used to construct Tobacco rattle virus (TRV)-based VIGS vectors. The resultant vectors TRV-*MdHIR4*, TRV-*MdJAZ2* and their combination, TRV-*MdHIR4*+TRV-*MdJAZ2*, were used in the injection of the fruit, while the empty TRV vector was used as the control. **(E)** Quantitative real-time PCR analysis of the expression levels of the *MdCHS, MdCHI, MdMYB9, MdMYB11, MdMYB1, MdANR, MdUFGT, Md*b*HLH3, MdANS* and *MdFLS* genes in the fruit peel around the injection sites. Md18S was used as a loading control.

To examine if and how MdHIR4 influences the accumulation of anthocyanins in the apple fruit peel, a TRV(Tobacco Rattle Virus)-based VIGS technique was used to transiently suppress the expression of *MdHIR4* and *MdJAZ2* in the peel of bagged fruit immediately following their detachment from the apple tree. The plasmids TRV-*MdJAZ2*, TRV-*MdHIR4* or TRV-*MdJAZ2*+TRV-*MdHIR4* were injected into the fruit peel and treated the apple leaf. Quantitative real-time PCR analysis showed that the injection or treatment correspondingly suppressed the expression levels of the *MdJAZ2* and *MdHIR4* genes in the peel and leaf (Fig. 5A,B; Fig. S3A,B). However, the TRV-induced *MdJAZ2* suppression did not influence the expression of *MdHIR4*, while *MdHIR4* did not influence *MdJAZ2* (Fig. 5A and B; Fig. S3C).

As a result, the suppression of *MdJAZ2* or *MdHIR4* alone noticeably promoted anthocyanin accumulation and fruit/leaf coloration in the peel and leaf. A co-injection with TRV-*MdJAZ2*+TRV-*MdHIR4* resulted in the production of more anthocyanins and the redder color in the fruit peel and leaf than a single injection of either TRV-*MdJAZ2* or TRV-*MdHIR4* (Fig. 5C,D; Fig. S3C,D). Furthermore, the relative expression of the regulatory genes, *MdMYB1, MdMYB9* and *MdMYB11*, as well as the structural genes, *MdCHS, MdCHI, MdF3H, MdDFR, MdANS, MdUFGT, MdANR* and *MdFLS*, were determined through quantitative real-time PCR of the fruit peel around the injection sites. The results indicated that the suppression of *MdHIR4* and *MdJAZ2* remarkably enhanced the expression levels of these ten regulatory and structural genes (Fig. 5E).

**Figure 6:**
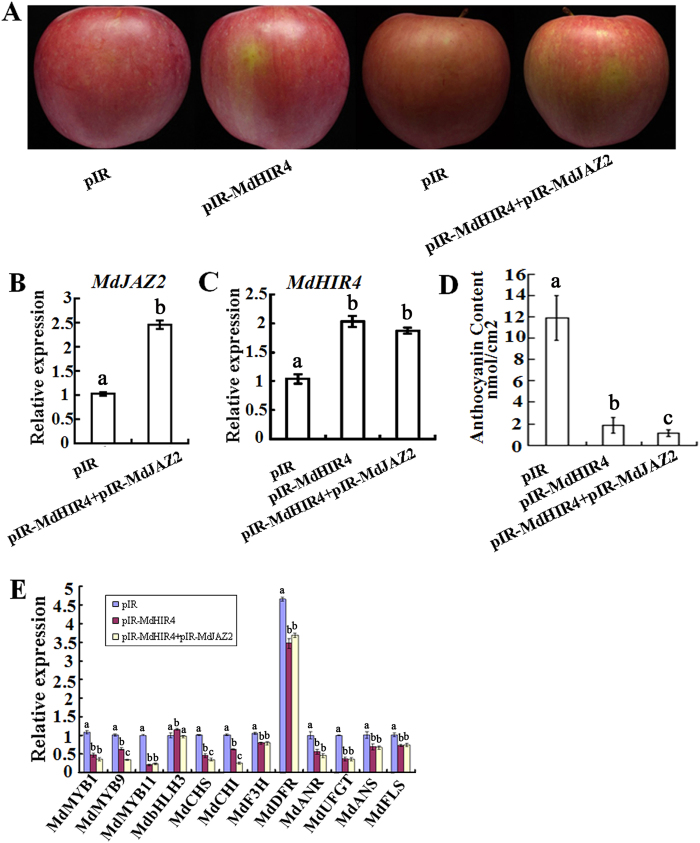
Overexpression of *MdHIR4* and *MdJAZ2* inhibits anthocyanin accumulation in apple peel. **(A)** The viral based fusion expression vectors pIR-*MdHIR4* and pIR-*MdHIR4*+pIR-*MdJAZ2* plus the helper plasmid IL-60-BS were injected into the apple fruit. The empty pIR vector plus the helper plasmid IL-60-BS was used as the control. The fruit peel coloration was observed 4 days after exposure to white light at 17 °C. **(B,C**) Quantitative real-time PCR analysis of the expression levels of *MdJAZ2* and *MdHIR4* in the fruit peels around the injection sites. **(D)** Anthocyanin content in the fruit peels around the injection sites. **(E)** qRT-PCR analysis of *MdMYB1, MdbHLH3, MdMYB9, MdMYB11, MdCHS, MdCHI, MdF3H, MdDFR, MdANR, MdUFGT, MdANS* and *MdFLS* in the apple fruit peels around the injection sites. *Md18S* was used as a loading control.

Additionally, a viral vector-based transformation method was carried out to enhance the expression of the *MdHIR4* and *MdJAZ2* genes. The plasmids pIR, pIR-*MdHIR4* and pIR-*MdJAZ2*+pIR-*MdHIR4* were injected into the fruit peel. The results showed that MdHIR4 negatively regulated anthocyanin accumulation and peel coloration in the apple fruit (Fig. 6).

## Discussion

Plants produce anthocyanins in almost all organs, such as fruit, flowers and leaves, in response to the ripening and maturation processes, and in response to biotic and abiotic stresses due to its antioxidant activity^37^,^38^. As is well known, jasmonic acid (JA) is an important phytohormone that induces anthocyanin biosynthesis via the JA signaling pathway. On the contrary, JAZ proteins interact with the WD40/bHLH/MYB complex and act as negative regulators for anthocyanin accumulation in various plant species^15^,^34^. In this study, we identified the MdJAZ-interacting proteins, the MdHIRs, through a yeast two-hybrid screen and then functionally characterized and identified them as negative regulators of anthocyanin biosynthesis in apple.

The HIR proteins ubiquitously exist in plant species. In *Arabidopsis*, there are four HIR genes, *HIR1, HIR2, HIR3* and *HIR4*, in the genome. Similarly, in the apple genome there are five MdHIR genes, *MdHIR1-1, MdHIR1-2, MdHIR2, MdHIR3* and *MdHIR4* (Fig. 2). Additionally, the HIR genes have been cloned and identified in various plants, such as tobacco, maize, barley, rice, wheat, pepper and legumes. In these plant species, the HIR proteins contain highly conserved amino acid sequences, and possess the SPFH domain (also named the PHB domain)^39^. They belong to a protein superfamily that controls cell proliferation, ion channel regulation and cell death^40^,^41^.

In this study, it was found that the PHB domain of MdHIR2 and MdHIR4 interacted with the N-terminal ZIM domain of the MdJAZ proteins (Fig. 3E and F). The ZIM (also named the TIFY) domain primarily mediates the homo- and heteromeric interactions between most of the JAZs in *Arabidopsis* and is involved in JA signaling output^42^. In addition, it also binds to Novel Interactor of JAZ (NINJA), which contains an EAR motif, and mediates the interaction between the JAZ proteins and TOPLESS to negatively regulate JA signaling^43^. Jasmonate does not affect the stability of NINJA, and NINJA overexpression does not affect JAZ3 stability^43^. The MdHIR proteins interacted with the ZIM domain of the MdJAZ proteins and inhibited their degradation in apple (Figs 3C–F and 4D).

**Figure 7:**
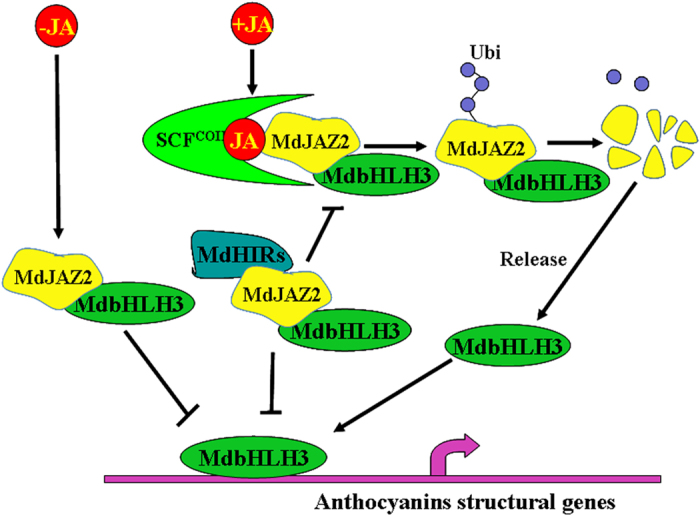
A model illustrating the negative regulation of anthocyanin accumulation by MdHIR4 through its interaction with and stabilization of the MdJAZs. When Jasmonate levels are low in the apple fruit the MdJAZ2 protein interacts with the MdbHLH3 protein and inhibits the promotion of anthocyanin structural genes. When Jasmonate levels are high in the apple fruit the MdJAZ2 protein is degraded by the SCF^COI1^ complex, which releases the transcription factor MdbHLH3. MdbHLH3 binds to the promoter of the anthocyanin structural genes and induces anthocyanin biosynthesis. However, the MdHIR proteins interact with the ZIM domain of the MdJAZ2 protein, but do not influence the interaction between MdbHLH3 and the Jas domain of the MdJAZ2 protein. Consequently, the MdHIR proteins inhibit the SCF^COI1^-induced degradation of the MdJAZ2 proteins, thereby strengthening the association of MdJAZ2 and MdbHLH3, and inhibiting the release of the MdbHLH3 protein. As a result, MdHIR4 negatively regulates anthocyanin biosynthesis and fruit coloration in the apple fruit.

In addition to the ZIM domain, the JAZ proteins also contain a C-terminal Jas motif that is required for the interactions between the JAZ proteins and COI1 and MYC2. The degradation of the JAZ proteins is induced by JA, and depends on the interaction between JAZ and the SCF^COI1^ protein complex^32^. Jasmonate induces anthocyanin accumulation by promoting the degradation of JAZ proteins in plants^15^,^34^. The MdHIR proteins inhibited the degradation of the MdJAZ proteins (Fig. 4C,D), thereby negatively regulating the biosynthesis of anthocyanins in apple. A model summarizing our findings regarding the regulatory pathway through which the MdHIR proteins inhibit the biosynthesis of anthocyanins is presented in Fig. 7. Additionally, the AtHIRs interacted with the AtJAZ proteins in *Arabidopsis* (Fig. S2), suggesting that this mechanism could work in other plant species.

In plants, the HIR proteins are involved in the hypersensitive response (HR). The HR is one of the various defense mechanisms mounted by plants in response to pathogen attack. This response is now almost universally accepted as a form of programmed cell death (PCD) characterized by the rapid death of plant cells at the site of pathogen infection. It generally causes localized cell death and results in necrotic lesions around infection sites in different plant organs. In barley and wheat, the *Hv-HIR1, Hv-HIR2, Hv-HIR3* and *Hv-HIR4* genes are induced by pathogens and are involved in the induction of the HR^44^,^45^. In *Arabidopsis*, the HIR proteins physically associate with the immune receptor RPS2 in plant immune responses^46^. In rice, a novel simple extracellular leucine-rich repeat (eLRR) domain protein, OsLRR1, enters the endosomal pathway and interacts with OsHIR1 to participate in PCD^47^. In wheat, the hypersensitive-induced reaction genes, *TaHIR1* and *TaHIR3*, play positive roles in resistance to the stripe rust fungus^48^. In pepper, CaLRR1 specifically binds to the plasma membrane (PM)-localized CaHIR1 to regulate PCD in leaves in response to an infection by *Xanthomonas campestris* pv. *Vesicatoria*^49^.

The HR is one of the most characteristic plant defenses against biotrophic pathogens. Salicylic acid (SA) plays a primary role in the activation of disease resistance mechanisms frequently associated with the HR^50^. The resistance (R) protein, which is a pathogen-encoded avirulence protein, can trigger the HR^51^. The R protein-mediated HR and SA-mediated basal resistance are generally considered effective against biotrophic pathogens, but ineffective against necrotrophic pathogens. Instead, plant resistance to necrotrophic pathogens is often mediated by jasmonic acid (JA) signaling, a process also involved in plant responses to wounding^52^.

During the HR, ROS production activates PCD^53^. Interestingly, anthocyanin works as an active scavenger of ROS in plant cells^54^. During PCD in lace plant (*Aponogeton madagascariensis*) leaves, the first visible change observed is the reduction of visible anthocyanin^55^, suggesting that anthocyanin may be involved in PCD. Therefore, the MdHIR-mediated inhibition of anthocyanin accumulation may be conducive to the occurrence of HIR and PCD.

Both isoflavonoid phytoalexin concomitants and anthocyanins are biosynthesized through the phenylpropanoid metabolic pathway. They belong to two different branches of this pathway, thereby being theoretically competitive with each other. For example, there in soybean is a strong bias towards decreasing the synthesis of anthocyanins and proanthocyanins, but increasing the synthesis of isoflavonoid phytoalexin concomitants during the resistance response^56^. It seems reasonable to suppose that the MdHIR-mediated inhibition of anthocyanin and proanthocyanin accumulation promotes the production of isoflavonoid phytoalexin concomitants, which may be conducive to PCD and HR. In addition, MdHIR proteins stabilize MdJAZ2 proteins (Fig. 4C,D), while JA promotes the degradation of the JAZ proteins. Therefore, JA should inhibit the function of the MdHIR proteins. As a result, JA seems to restrict cell death processes associated with hypersensitive induced reaction (HIR) in response to SA, ethylene, and ROS^57^, and thus is part of the machinery that prevents excessive damage to host tissues.

## Material and Methods

### Plant materials

Apple fruits (*Malus domestica* Borkh.) were bagged at 30 days after full bloom (DAFB). The bagged fruits of ‘Red Delicious’ cultivar were harvested from a adult tree at 140 DAFB, while those of ‘Fuji’ at 170 DAFB. After injection, fruit was placed in an illumination incubator for three days at 17 °C. The callus of the ‘*Orin*’ cultivar was grown on MS medium containing 1.5 mg L^−1^ 6-benzylaminopurine (6-BA) and 0.5 mg L^−1^ indole-3-acetic acid (IAA) at 25 °C in the dark. The tissue cultures of the ‘Gala’ cultivar *in vitro* were cultured on MS medium supplemented with 0.2 mg L^−1^ IAA and 1.5 mg L^−1^ 6-BA at 25 °C under a 16-h light/8-h dark photoperiod.

### Determination of total anthocyanins

Anthocyanins were extracted from 8 pieces of apple skin, each of 1 cm^2^ in size from one individual fruit, in 1 mL 1% (v/v) HCl-methanol for 24 h at room temperature in the dark. The upper aqueous phase was separated by centrifugation for 5 min at 13000 g and was subjected to spectrophotometric quantification at 650, 620 and 530 nm using a UV–Vis spectrophotometer (Shimadzu UV-2450, Kyoto, Japan). The content of the anthocyanins was determined using the following formula: OD = (A530–A620)–0.1 (A650–A620)^58^.

### Gene cloning and expression analysis

Total RNA was extracted from plant material for gene cloning and expression analyses using TRIzol reagent (Invitrogen, Carlsbad, CA, USA). For quantitative real-time PCR analyses, we used the clone enzyme Kit (Transgene, Beijing, China) and SYBR Green MasterMix (SYBR Premix EX Taq TM, Dalian, China), according to the manufacturer’s instructions. The primer sequences used for quantitative real-time PCR analyses are listed in Supplemental Table S1.

### DNA constructs and genetic transformation

The full length coding regions of *MdJAZ2* and *MdHIR4* were amplified from the cDNA of *Malus domestica* using standard molecular biology protocols and enzymatic digestion technology (Invitrogen, Carlsbad, CA, USA). The *MdHIR4* PCR product was recombined with vector pCXSN-HA^59^ to create the pCXCN-*MdHIR4* plasmid. The *MdJAZ2* PCR product was cloned into pMD18-T (Takara Bio, Atsu, Japan) to create a fusion protein of the *GUS* gene and the coding region of *MdJAZ2*, for convenient detection of MdJAZ2. The *MdJAZ2-GUS* fusion was recombined into the vector pRI101-AN (Takara Bio, Atsu, Japan), to form the pRI-*MdJAZ2*-*GUS* plasmid. In both expression vectors the promoter was the cauliflower mosaic virus (CaMV) *35S* promoter. All the specific primers and restriction enzymes used are listed in Supplemental Table 1. The two plasmids were introduced into the *Agrobacterium tumefaciens* strain LBA4404, and the resultant *Agrobacterium tumefaciens* transformants were used to transform the apple callus as described by An *et al*.^18^.

### Yeast two-hybrid (Y2H) screening and assays

To screen for proteins that interact with MdJAZ2, the full-length CDS of the *MdJAZ2* gene was inserted into pGBT9 vector which contain the DNA binding domain of GAL4. The resulting construct, MdJAZ2-BD, was used as bait vector to screen an apple cDNA library. The cDNA library was made with total RNAs isolated from apple fruit peel and constructed by Oebiotech Company (Shanghai, China).

Y2H assays were carried out as described by Xie *et al*. (2012). All of the coding regions of the *JAZ* genes used in this study were amplified from the cDNA of *Arabidopsis* and *Malus domestica.* The *JAZ* genes were recombined with the vector pGAD424 to generate constructs containing the *JAZ* genes fused to the GAL4 activation domain for Y2H analysis. The coding regions of the *HIR* genes used in this study were amplified from the cDNA of *Arabidopsis* and *Malus domestica.* They were then recombined with the vector pGBT9 to generate constructs of the *HIR* gene fused to the GAL4 DNA binding domain for Y2H analysis. The specific primers and restriction enzymes that were used are listed in Supplemental Table 1.

Yeast transformants were exhaustively screened on synthetic defined (SD) media (-Leu/-Trp/-His/-Ade) according to the manufacturer’s instructions (Clontech, Palo Alto, CA, USA). The *JAZ*-AD and *HIR*-BD plasmids were co-transformed into the Y2HGOLD yeast cell strain using the lithium acetate method and were cultured at 30 °C. The resulting yeast transformants were filtered on medium lacking Trp and Leu (-Trp/-Leu), and putative transformants were subsequently transferred to medium lacking Trp, Leu, His and Adenine (-Leu/-Trp/-His/-Ade) with and without X-alpha-gal.

### Pull-down assays

The full-length coding sequences of *MdJAZ2, AtJAZ3, AtJAZ9* and *MdHIR2, MdHIR4, AtHIR1* were cloned into the pGEX-4T-1 and pET32a vectors, respectively, to generate the *JAZ*-HIS and *HIR*-GST constructs used for the pull-down assays. All the specific primers and restriction enzymes that were used are listed in Supplemental Table 1.

For the *in vitro* pull-down experiments, expression of the proteins MdJAZ2/AtJAZ3/AtJAZ9-HIS and MdHIR2/MdHIR4/AtHIR1-GST was induced and the proteins were purified from *E. coli* BL21 cells. The purified protein mixtures of the JAZ and HIR genes were incubated for 1 h at 4 °C, 80 rpm, and purified using a His purification kit (Cwbio, Beijing, China), according to the manufacturer’s instructions. Finally, the proteins were detected through Western blot analysis using anti-His and anti-GST antibodies.

### Construction of the viral vectors and the Agrobacterium infiltration of the apple fruit

Viral vectors were used as described by Li *et al*.^20^. to observe the effects of *MdJAZ2* and the *MdHIR* genes overexpression or suppression in apple fruit. The full-length sequences of *MdJAZ2* and *MdHIR4* were cloned into the pIR vector under the control of the *35S* promoter. The overexpression constructs were named pIR*-MdJAZ2* and pIR*-MdHIR4*. To generate antisense expression vectors for *MdJAZ2* and *MdHIR4*, the fragment of *MdJAZ2* and *MdHIR4* used in the above-mentioned genetic transformation was cloned into the tobacco rattle virus (TRV) vector in an antisense orientation under the control of the *35S* promoter. All the specific primers and restriction enzymes that were used are listed in Supplemental Table 1. The resultant vectors were named TRV*-MdJAZ2*, and TRV*-MdHIR4*. The resultant viral vectors were used for injection into the apple fruit as described by Xie *et al*.^12^.

### The degradation of the protein

The callus of the ‘*Orin*’ cultivar, which was transformed with the pRI-*MdJAZ2*-*GUS* fusion gene, was grown on the culture medium for 15 days and treated with 50 mM MG132 (proteinase inhibitor, Sigma-Aldrich) overnight in the dark. Then, the calli were washed clean and treated with 50 μM Me-JA. Untreated calli was used as a control. The JAZ protein was detected through Western blotting using an anti-GUS antibody (BGI, Beijing, China). The ‘*Orin*’ callus co-transformed with *35S::MdJAZ2*-*GUS* and *35S::MdHIR4* was obtained and used to testing for the function of the HIR protein in regards to the stability of JAZ. The amount of JAZ protein was detected in the *35S::MdJAZ2*-*GUS* callus and compared with the amount of JAZ protein in the *35S::MdJAZ2-GUS*+*35S::MdHIR4* callus by means of Western blotting using the anti-GUS tag antibody (BGI, Beijing, China).

## Additional Information

**How to cite this article:** Chen, K.-Q. *et al*. MdHIR proteins repress anthocyanin accumulation by interacting with the MdJAZ2 protein to inhibit its degradation in apples. *Sci. Rep.* **7**, 44484; doi: 10.1038/srep44484 (2017).

**Publisher's note:** Springer Nature remains neutral with regard to jurisdictional claims in published maps and institutional affiliations.

## Supplementary Material

Supplementary Datasets
