# Supplementary material for: MdHIR proteins repress anthocyanin accumulation by interacting with the MdJAZ2 protein to inhibit its degradation in apples
Source: Sci Rep. 2017 Mar 20;7:44484. doi: 10.1038/srep44484 (PMC5357849; doi:10.1038/srep44484)
Supplement: Supplementary Datasets [file srep44484-s1.doc]

**Supplementary data**

MdHIR proteins repress anthocyanin accumulation by interacting with the MdJAZ2 protein to inhibit its degradation in apples

Ke-Qin Chen^1^, Xian-Yan Zhao^1^, Xiu-Hong An, Yi Tian, Dan-Dan Liu, Chun-Xiang You*, Yu-Jin Hao*

**Supplementary Figure S1**. The PCR results of the five MdHIRs genes from the Reverse transcription PCR analysis.


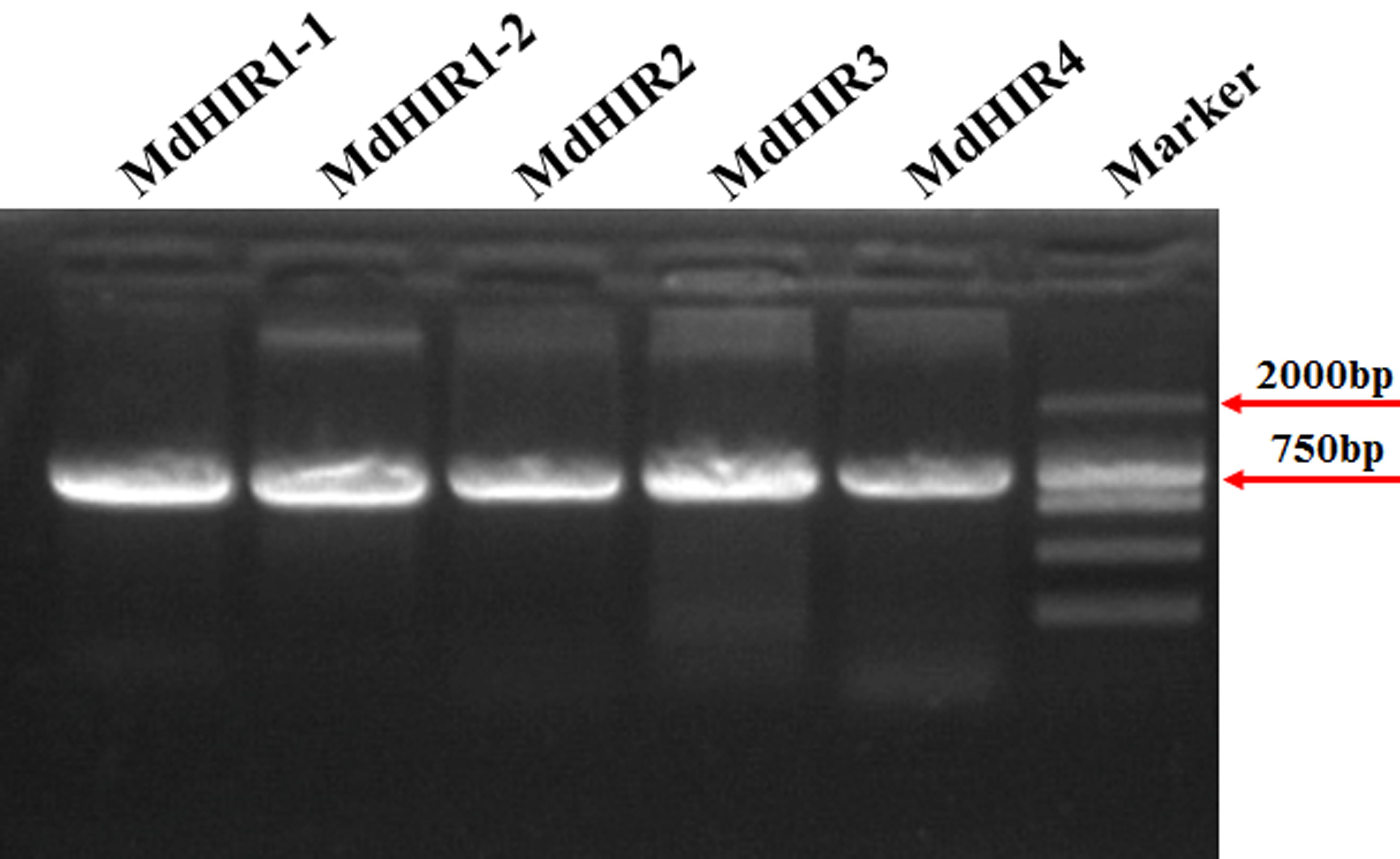


**Supplementary Figure S2**. The interaction between the AtJAZs and the AtHIRs in Arabidopsis. **(A)** Yeast two-hybrid assays of the interactions between the AtJAZs and the AtHIRs. Each of the 12 AtJAZ proteins were individually fused with the GAL4 activation domain (AD) in the pGAD424 vector, while each of the 4 AtHIR proteins were fused with the GAL4 DNA binding domain (BD) in the pGBT9 vector. Interactions of the AtHIRs with the AD domain in the empty pGAD424 vector were used as the negative control. Interactions are indicated by the blue color of the yeast on SD/-Ade/-His/-Trp/-Leu/X-Gal medium. **(B-C)** In vitro pull-downs to assay the interaction of AtJAZ3-HIS and AtJAZ9-HIS with HIR1-GST. Purified recombinant Arabidopsis JAZ3 and JAZ9 protein (fused to a 6xHis tag) was used to pull down AtHIR1 protein (fused to the GST tag). As a control, purified recombinant 6xHis protein did not pull down AtHIR1-GST.


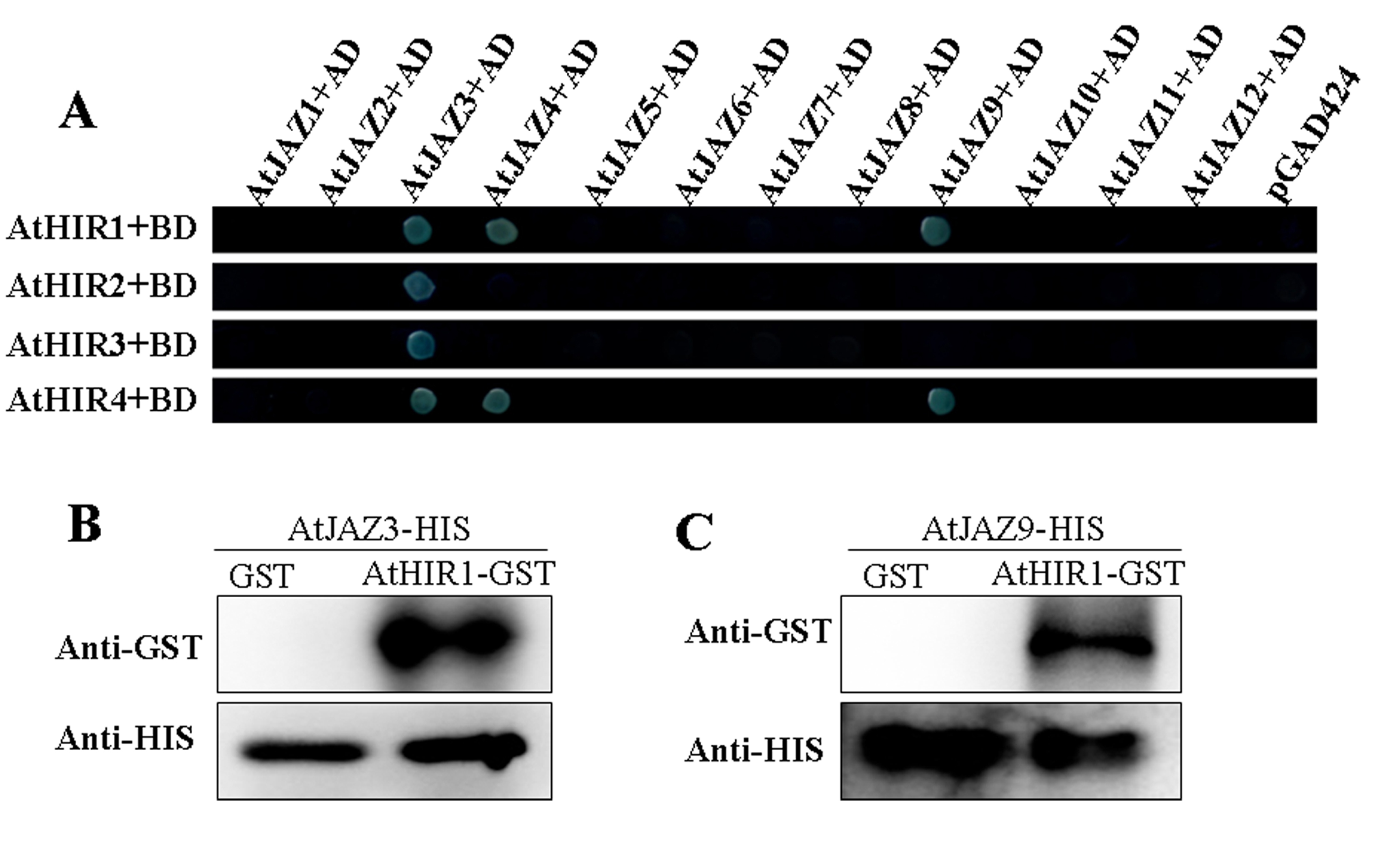


**Supplementary Figure S3.** The transient expression experiment of antisense of MdHIR4 and MdJAZ2 enhanced anthocyanin accumulation in apple leaves. **(A-B)** The expression level of MdJAZ2 and MdHIR4 in apple leaves which were treated with TRV-MdHIR4, TRV-MdJAZ2 and TRV-MdHIR4 plus TRV-MdJAZ2, the empty TRV vector was used as the control. **(C)** The anthocyanin content in the treated apple leaves. **(D)** The coloration in the treated apple leaves. E. The anthocyanin was extracted from the treated apple leaves.


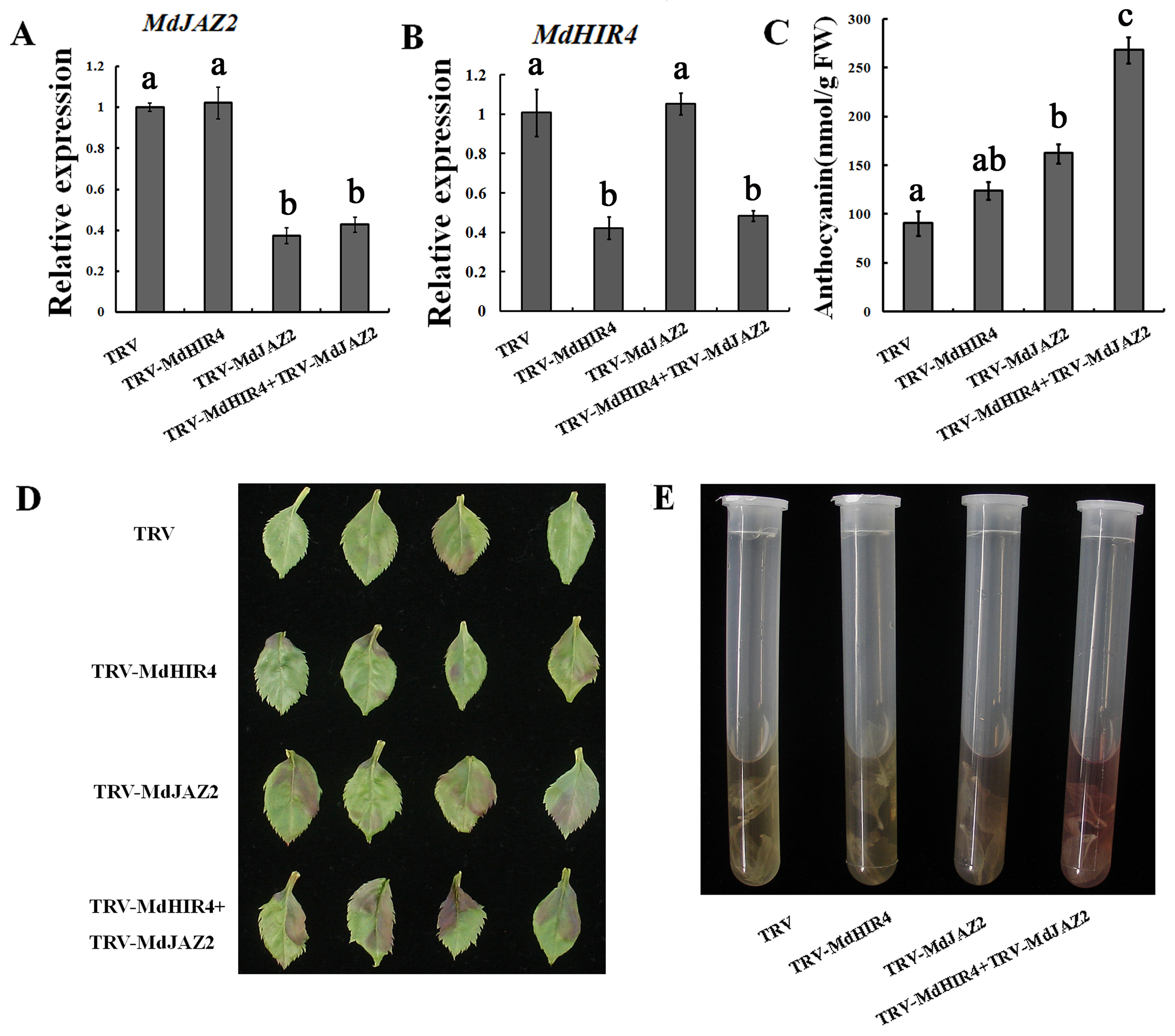


**Supplement table1 List of primers used in this research.**

| Gene name | Enzymes | Primer sequence(5'→3') |
| --- | --- | --- |
| IL60-MdJAZ2 | *Sal*Ⅰ, *Xba*Ⅰ | F: GTCGACTACCCATACGATGTTCCAGATTACGCTATGGAGAGAGATTTCTTGGGT  R: TCTAGA TCACCTTTCCTTGCGCTTCTC |
| IL60-MdHIR4 | *Sal*Ⅰ, *Xba*Ⅰ | F: GTCGACCATCATCATCATCATCATATGAGCAATTCGTGCTGCTGC  R: TCTAGA TTACTCGGCATTTAGCTGAGC |
| TRV-MdJAZ2 | *Eco*RⅠ, *Bam*HⅠ | F: GAATTCcgggataactgttattttcta  R: GGATCCttggttcggtttttggagaag |
| TRV-MdHIR4 | *Eco*RⅠ, *Bam*HⅠ | F: GAATTCGTGAAGCCCACTTTGTTAACT  R: GGATCCGTGATCAAATACGCAACGGAC |
| pGBT9-MdHIR1-1 | *Bam*HⅠ, *Sal*Ⅰ | F: GGATCCGG ATGGGTAATTTATTCTGTTGT  R: GTCGAC CTACTGGTGAGAACCCTGAAG |
| pGBT9-MdHIR1-2 | *Bam*HⅠ, *Sal*Ⅰ | F: GGATCCGGATGGGTAATCTATTCTGTTGT  R: GTCGAC CTACTGATAAGAACCCTGAAG |
| MdHIR4-N | *Bam*HⅠ, *Sal*Ⅰ | F: GGATCC TGAGCAATTCGTGCTGCTGC  R: GTCGACgtatacgttggcaagctggag |
| MdHIR4-C | *Bam*HⅠ, *Sal*Ⅰ | F: GGATCCgcagctcaaaggctccagctt  R: GTCGAC TTACTCGGCATTTAGCTGAGC |
| pGBT9-MdHIR2 | *Bam*HⅠ, *Sal*Ⅰ | F: GGATCCATGGGACAAGCTCTTGGTTGT  R: GTCGACTTACTCCTGAATCGAACTTCC |
| pGBT9-MdHIR3 | *Bam*HⅠ, *Sal*Ⅰ | F: GGATCCTGGGTCAAGCACTTGGTTGT  R: GTCGACTTATTCCTTAGCATAATCGGC |
| pGBT9-MdHIR4 | *Bam*HⅠ, *Sal*Ⅰ | F: GGATCCTGAGCAATTCGTGCTGCTGC  R: GTCGACTTACTCGGCATTTAGCTGAGC |
| pGBT9-MdHIR2-N | *Bam*HⅠ, *Sal*Ⅰ | F: GGATCCATGGGACAAGCTCTTGGTTGT  R: GTCGACTGCATTAATCTCATTCATCGA |
| pGBT9-MdHIR2-C | *Bam*HⅠ, *Sal*Ⅰ | F: GGATCCGCTGCTAGGATGAGGGTGGCC  R: GTCGACTTACTCCTGAATCGAACTTCC |
| pGBT9-AtHIR1 | *Eco*RⅠ, *Sal*Ⅰ | F: GAATTCatggggaatttgttttgt  R: GTCGACtcacaggtttgcggacga |
| pGBT9-AtHIR2 | *Eco*RⅠ, *Sal*Ⅰ | F: GAATTCatgggtcaagctttgggt  R:GTCGACctactcagcagcagagtt |
| pGBT9-AtHIR3 | *Eco*RⅠ, *Sal*Ⅰ | F:GAATTCatggggaatcttttctgt  R:GTCGACttaggaggcattgttggc |
| pGBT9-AtHIR4 | *Eco*RⅠ, *Sal*Ⅰ | F:GAATTCatgggtaatacgtattgt  R:GTCGACctagacgtcgttgacctg |
| pGAD424-MdJAZ1 | *Bam*HⅠ, *Sal*Ⅰ | F:GGATCCTGGAGAGAGATTTCTTGGGC  R:GTCGACTCATCGTTGTTCTCCTTGTTG |
| pGAD424-MdJAZ2 | *Eco*RⅠ, *Sal*Ⅰ | F: GAATTCATGGAGAGAGATTTCTTGGGT  R:GTCGACTCACCTTTCCTTGCGCTTCTC |
| pGAD424-MdJAZ3 | *Eco*RⅠ, *Sal*Ⅰ | F: GAATTCATGTCGAGTTCGTCGGAGACT  R: GTCGACTCATTGGGTTTGCTGAGCAGC |
| pGAD424-MdJAZ4 | *Bam*HⅠ, *Sal*Ⅰ | F: GGATCCTGCAGTGGTCGTTTTCAAAC  R: GTCGACTCAGATTGAGCCTGCATGTTG |
| pGAD424-MdJAZ5 | *Eco*RⅠ, *Sal*Ⅰ | F: GAATTCATGGCAGCTGAGAAGTTGAAT  R: GTCGACCTAGAGGCTGAGTTCAAGCTG |
| pGAD424-MdJAZ6 | *Eco*RⅠ, *Sal*Ⅰ | F: GAATTCATGGAGGAAAAAGCAGAAGTT  R: GTCGACCTAGGCAACCGAACTTGGTTG |
| pGAD424-MdJAZ8 | *Sma*Ⅰ, *Sal*Ⅰ | F: cccgggcATGCCCAGAGCCACCGTTGAG  R: gtcgacTTAGGTTTGGCAGGCATATGG |
| pGAD424-MdJAZ2-1 | *Eco*RⅠ, *Sal*Ⅰ | F: GAATTCATGGAGAGAGATTTCTTGGGT  R: GTCGACTCACCTTTCCTTGCGCTTCTC |
| pGAD424-MdJAZ2-2 | *Eco*RⅠ, *Sal*Ⅰ | F: GAATTCACCTCTGGGTCACCTTCACAA  R: GTCGACAGCACTAGAGGAGTTAGAACA |
| pGAD424-AtJAZ1 | *Eco*RⅠ, *Sal*Ⅰ | F: GAATTCATGTCGAGTTCTATGGAATGT  R: GTCGACTCATATTTCAGCTGCTAAACC |
| pGAD424-AtJAZ2 | *Eco*RⅠ, *Sal*Ⅰ | F: gaattcATGTCGAGTTTTTCTGCCGAG  R: gtcgacTTACCGTGAACTGAGCCAAGC |
| pGAD424-AtJAZ3 | *Eco*RⅠ, *Sal*Ⅰ | F: gaattcATGGAGAGAGATTTTCTCGGG  R: gtcgacTTAGGTTGCAGAGCTGAGAGA |
| pGAD424-AtJAZ4 | *Eco*RⅠ, *Sal*Ⅰ | F: gaattcATGGAGAGAGATTTTCTCGGG  R: gtcgacTTAGTGCAGATGATGAGCTGG |
| pGAD424-AtJAZ5 | *Eco*RⅠ, *Sal*Ⅰ | F: gaattcATGTCGTCGAGCAATGAAAAT  R: gtcgacCTATAGCCTTAGATCGAGATC |
| pGAD424-AtJAZ6 | *Eco*RⅠ, *Sal*Ⅰ | F: gaattcATGTCAACGGGACAAGCGCCG  R: gtcgacCTAAAGCTTGAGTTCAAGGTT |
| pGAD424-AtJAZ7 | *Eco*RⅠ, *Sal*Ⅰ | F: gaattcATGATCATCATCATCAAAAAC  R: gtcgacCTATCGGTAACGGTGGTAAGG |
| pGAD424-AtJAZ8 | *Sma*Ⅰ, *Sal*Ⅰ | F: cccgggaATGAAGCTACAGCAAAATTGT  R: ctgcagTTATCGTCGTGAATGGTACGG |
| pGAD424-AtJAZ9 | *Bam*HⅠ, *Sal*Ⅰ | F: GGATCC TGGAAAGAGATTTTCTGGGT  R: AAGCTT TGTAGGAGAAGTAGAAGAGTA |
| pGAD424-AtJAZ10 | *Eco*RⅠ, *Sal*Ⅰ | F: gaattcATGTCGAAAGCTACCATAGAA  R: gtcgacTTAGGCCGATGTCGGATAGTA |
| pGAD424-AtJAZ11 | *Eco*RⅠ, *Sal*Ⅰ | F: gaattcATGGCTGAGGTAAACGGAGAT  R: gtcgacTCATGTCACAATGGGGCTGGT |
| pGAD424-AtJAZ12 | *Sma*Ⅰ, *Sal*Ⅰ | F: cccgggaATGACTAAGGTGAAAGATGAG  R: ctgcagCTAAGCAGTTGGAAATTCCTC |
| pGEX-4T-1-MdHIR2 | *Bam*HⅠ, *Sal*Ⅰ | F: ggatccATGGGACAAGCTCTTGGTTGT  R: gtcgacTTACTCCTGAATCGAACTTCC |
| pGEX-4T-1-MdHIR4 | *Bam*HⅠ, *Sal*Ⅰ | F: GGATCCATGAGCAATTCGTGCTGCTGC  R: GTCGACTTACTCGGCATTTAGCTGAGC |
| pGEX-4T-1-AtHIR1 | *Bam*HⅠ, *Sal*Ⅰ | F: GGATCCatggggaatttgttttgt  R: GTCGACtcacaggtttgcggacga |
| pET32a-MdJAZ2 | *Eco*RⅠ, *Sal*Ⅰ | F: GAATTCATGGAGAGAGATTTCTTGGGT  R: GTCGACTCACCTTTCCTTGCGCTTCTC |
| pET32a-AtJAZ3 | *Eco*RⅠ, *Sal*Ⅰ | F: gaattcATGGAGAGAGATTTTCTCGGG  R: gtcgacTTAGGTTGCAGAGCTGAGAGA |
| pET32a-AtJAZ9 | *Bam*HⅠ, *Sal*Ⅰ | F: GGATCC ATGGAAAGAGATTTTCTGGGT  R: AAGCTT TGTAGGAGAAGTAGAAGAGTA |
| pCXSN- MdHIR4-HA |  | F: AATGAGCAATTCGTGCTGCTGC  R: TTACTCGGCATTTAGCTGAGC |
| pRI101-MdJAZ2-GUS | *Sal*Ⅰ, *Bam*HⅠ | F: GTCGACATGGAGAGAGATTTCTTGGGT  R: GAATTCTCACCTTTCCTTGCGCTTCTC |
| MdPAL(RT) |  | F: gtgagggaggagttgggaggag  R: ctcctcccaactcctccctcac |
| MdCHI(RT) |  | F: gctacaaatgcggtgatag  R: CGCCTCCACTACAACCTCC |
| MdCHS(RT) |  | F: ggcaagtgctgtcggatt  R: CCCAAAGAAATAACCACAAG |
| MdF3H(RT) |  | F : GCCGATCACCTACACCGAG  R : GTACAAGAAGTGGGAAGGC |
| MdDFR(RT) |  | F: gttgagggagatagggtttgag  R: GGTAAATGTAAAACAATAGAGAGG |
| MdANR（RT） |  | F: TCAACAAAAGATACCCCCAG  R: gatagctagctcgatacatgc |
| MdUFGT(RT) |  | F: GGAAGTGGTTTTGTCGCCTG  R: CATTATTATTGAGCAACGAACAGC |
| MdANS(RT) |  | F: ggagaagatcatccttaagcca  R: ctaagatatatcataccaactatgcc |
| MdFLS(RT) |  | F: ggataagacaagaatctcatggc  R: cacaccactcacaactttacc |
| MdHIR4(RT) |  | F: CTTCAAGTGAAAAGAGCAGAA  R: ACCATGCGGTAGAAAAACTGT |
| MdJAZ2(RT) |  | F: GGCAACGTTATATGCATTC  R: AGCCGTATGCTTCATGGTAGTT |
| MdMYB1(RT) |  | F: gaaagagctgcatatcccag  R: CTATTCTTCTTTTGAATGATTCC |
| MdMYB9(RT) |  | F: gatgaggcaatgataaatgacg  R: ttagactacaacattttcttggg |
| MdMYB11(RT) |  | F: GTCGATTTCTCTGTGCTCTATAAC  R: ttaattatctacgagccagcagtc |
| MdbHLH3（RT） |  | F: ccaaaaatggctgcaccgcc  R: cttgatagctccattatagtaccc |
